# Transcutaneous Vagus Nerve Stimulation at Olfactory Bulb‐Relevant Frequencies Modulates Human Olfactory Function

**DOI:** 10.1111/psyp.70408

**Published:** 2026-09-22

**Authors:** Anja L. Winter, Andrea Aejmelaeus‐Lindström, Kristen Gregory, Gregory Francis, Cristina Jaen, Pamela Dalton, Evelina Thunell, Artin Arshamian, Johan N. Lundström

**Affiliations:** ^1^ Department of Clinical Neuroscience Karolinska Institutet Stockholm Sweden; ^2^ Monell Chemical Senses Center Philadelphia Pennsylvania USA; ^3^ Department of Psychology University of Southern Denmark Esbjerg Denmark; ^4^ Department of Otorhinolaryngology Karolinska University Hospital Stockholm Sweden

**Keywords:** odor discrimination, olfactory function, sensory rehabilitation, tVNS, vagus nerve

## Abstract

Transcutaneous vagus nerve stimulation (tVNS) is a noninvasive technique that engages central neuromodulatory pathways and has been shown to modulate sensory processing across multiple modalities. Here, we investigated whether tVNS influences human olfactory perception. Sixty‐seven healthy adults were assigned to one of two stimulation frequency groups, 25 Hz (beta) or 55 Hz (gamma), and each received 20 min of tVNS applied to the left cymba concha as well as a control condition with identical stimulation delivered to the left earlobe. Odor discrimination, odor intensity, and visual attention were assessed before and after stimulation. Gamma frequency tVNS significantly decreased odor discrimination performance, whereas beta frequency did not. Odor intensity ratings increased modestly following stimulation in both groups, with a stronger effect observed after gamma‐frequency tVNS. There were no changes in visual attention, and the stimulation intensity, which was selected by the participant, was not associated with olfactory outcomes. These findings demonstrate that tVNS can modulate human olfactory processing in a frequency‐dependent manner. The selective effect of gamma frequency, that is, the known processing frequency of the human olfactory bulb, implies inhibitory interneuron engagement in the olfactory bulb. These findings demonstrate that noninvasive tVNS can modulate human olfactory processing in a frequency‐specific manner and highlight stimulation frequency as a critical parameter for future translational applications.

## Impact Statement

Whether stimulation of the human vagus nerve can modulate an olfactory bulb‐dependent odor task has remained untested. We show that transcutaneous vagus nerve stimulation at gamma frequency, the olfactory bulb's intrinsic oscillatory rhythm, selectively alters odor discrimination, while beta‐frequency stimulation has no effect. These findings establish a functional vagus–olfactory bulb pathway in humans and identify stimulation frequency as a key variable for developing noninvasive olfactory rehabilitation strategies.

Accurate odor discrimination depends on the olfactory bulb (OB), where the coordinated activity of excitatory mitral and tufted cells and inhibitory periglomerular and granule cell interneurons transform raw sensory input into refined odor representations (Mainland et al. 2014; Nagayama et al. 2014). As the brain's first central processing stage for olfactory information, the OB thus plays a critical role in determining the precision with which odors are distinguished. This processing is not static; the OB's inhibitory circuits are subject to modulation by descending cortical projections as well as ascending neuromodulatory systems that can alter the balance of excitation and inhibition and thereby change odor processing efficiency (Cleland and Linster 2005). Vagus nerve stimulation (VNS) engages several of these ascending systems through its projections to the nucleus tractus solitarius (NTS), with downstream activation of the locus coeruleus and other brainstem nuclei that project diffusely to forebrain structures (Rodenkirch et al. 2022). VNS has been shown to modulate sensory processing across multiple modalities, enhancing somatosensory processing through optimization of thalamocortical dynamics, increasing the amplitude of auditory event‐related potentials and the perceived intensity of gustatory stimuli, and improving task performance. Whether VNS can similarly modulate olfactory processing in humans, and through what mechanism, remains an open question.

The first evidence for a functional connection between the vagus nerve and the olfactory system came from animal electrophysiology, in which stimulation of the cervical vagus nerve in rats excited inhibitory interneurons within the periglomerular layers of the OB, producing decreased discharge rates in approximately half of recorded neurons (García‐Díaz et al. 1984). This finding established that vagus afferents, relayed through the NTS, can engage the OB's inhibitory circuitry and thereby alter local processing. Although no established monosynaptic connection exists between the vagus nerve and the OB in humans, VNS induces neuronal activity changes in structures with monosynaptic connections to the OB, such as the amygdala, hippocampus, and hypothalamus. Indeed, stimulation of the cymba conchae of the external ear, which is innervated by the auricular branch of the vagus nerve, activates the NTS in humans as demonstrated by functional neuroimaging (Frangos et al. 2015). Transcutaneous VNS (tVNS) applied to the cymba conchae therefore represents a potential noninvasive route for engaging vagus‐OB pathways in humans.

The effects of VNS on olfactory function appear to be highly dependent on stimulation frequency. In the original rodent work, only stimulation around 80 Hz reliably altered OB neuron firing, whereas frequencies of 20–40 Hz did not (García‐Díaz et al. 1984). This pattern has been partially replicated in humans, where noninvasive VNS stimulation with a frequency of 80 Hz improved supra‐threshold olfactory performance and increased orbitofrontal cortex activation in healthy adults, while stimulation of 10 Hz produced no measurable effect (Maharjan, Wang, et al. 2018). In line with this frequency dependence, implanted VNS at standard clinical frequencies (20–30 Hz) has generally failed to produce behavioral changes in olfactory measures, although electrophysiological changes in olfactory event‐related potentials have been observed (Kirchner et al. 2004; Sperling et al. 2011). Together, these findings identify stimulation frequency as a critical parameter and suggest that the frequencies capable of engaging OB circuits may correspond to the intrinsic oscillatory dynamics of the targeted neural populations. The human OB displays prominent oscillatory activity in the gamma band during active odor processing (Iravani et al. 2020, 2021; Nordén et al. 2024), and beta‐band oscillations have been associated with top‐down modulation and feedback processing (Kay and Beshel 2010; Nordén et al. 2024; Yang et al. 2022); whether tVNS at frequencies corresponding to these endogenous rhythms can selectively modulate OB function has not been tested.

Olfactory dysfunction is a common condition with limited treatment options; odor training, the primary evidence‐based intervention, produces modest effects and suffers from low patient compliance (Hummel et al. 2009; Winter et al. 2025). If tVNS can reliably modulate OB function, it could represent a novel complementary approach to olfactory rehabilitation. However, establishing whether and at what frequency tVNS affects the human OB is a necessary first step before any clinical application can be considered.

In the present study, we aim to determine whether tVNS modulates human olfactory bulb function, assessed through behavioral changes in odor discrimination performance.

Odor discrimination was selected as the primary outcome because causal rodent work links the ability to discriminate perceptually similar odors to inhibitory interneuron processing within the olfactory bulb (Abraham et al. 2010; Gschwend et al. 2015; Nunes and Kuner 2015; Yokoi et al. 1995). Although discrimination performance in humans also reflects contributions from attention, working memory, and perceptual decision‐making, these processes are common to the tVNS and control conditions in our within‐subject design and are further indexed by an independent visual attention task. Based on the established effects of VNS on inhibitory interneurons in the rodent olfactory bulb (García‐Díaz et al. 1984), we hypothesize that tVNS will modulate olfactory bulb activity in humans, resulting in measurable changes in odor discrimination ability. We further expect this effect to be frequency‐dependent, with high‐frequency stimulation producing more pronounced changes in discrimination performance compared to low‐frequency or sham stimulation conditions (Maharjan, Wang, et al. 2018). In summary, our aim is to directly test vagus‐olfactory interactions in humans using a noninvasive tVNS protocol, to provide functional evidence as to whether the human olfactory bulb can be influenced by vagus nerve activity.

Olfactory performance was assessed after, rather than during, stimulation. This design choice serves two purposes: it avoids the somatosensory and arousal‐related confounds inherent in concurrent electrical stimulation during olfactory testing, and it ensures identical testing conditions before and after stimulation in both treatment conditions. It also defines the mechanistic scope of the study: our measures index after‐effects of the stimulation period rather than online modulation during ongoing stimulation, and these two approaches likely probe partially distinct mechanisms.

## 1 Methods

### 1.1 Participants

A total of 67 healthy participants aged 18–50 years participated in this study divided into two experiments. Experiment 1 involved 25 participants (mean age 34.0, SD ±8.9, 14 women), and Experiment 2 involved 42 participants (mean age 34.7, SD ±9.4, 20 women). Both experiments were identical in their procedures except for stimulation frequency. Experiment 1 enrolled fewer participants due to interruption by the COVID‐19 pandemic. Inclusion criteria were self‐proclaimed as generally healthy, non‐smoker, not pregnant, no acute allergy to specific odors, no history of psychotic or psychological disorder, no history of head trauma leading to unconsciousness, and no contraindicator for nerve stimulation (such as the use of a pacemaker or defibrillator, history of epilepsy, seizure disorders, cardiovascular issues, or ongoing dermatological problems on the external ear). In addition, participants were required to have a functional olfactory sense, assessed with the Sniffin’ Sticks identification test (see Measurements for procedure). In Experiment 1, participants identified a mean of 14.1 odors correctly (range 11–16), whereas in Experiment 2, the mean was 13.6 (range 10–16). All participants provided written informed consent prior to participation, and all aspects of the study were approved by the University of Pennsylvania Institutional Review Board (2022/833822).

### 1.2 tVNS Stimulation

**FIGURE 1:**
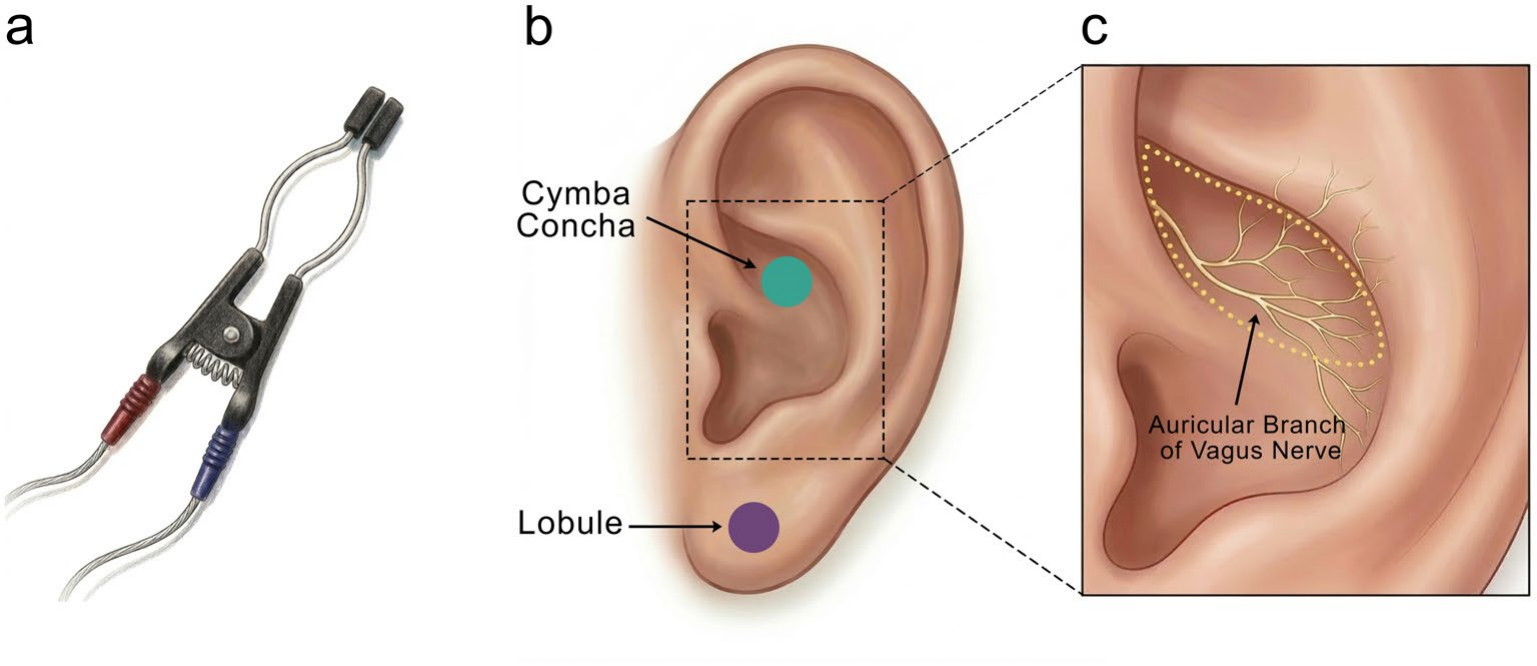
Stimulation method and area. (a) Stimulation electrode. (b) View of stimulation areas. Teal colored area in the cymba concha indicates area of stimulation for the tVNS condition and plum colored area of stimulation of the ear lobule for the Control condition. (c) Blow‐up box showing the auricular branch of the vagus nerve.

Each participant completed both tVNS stimulation (cymba concha area) and active control stimulation (earlobe) (Figure 1). To minimize potential cardiac effects (Borges et al. 2021; Forte et al. 2022), stimulation was delivered to the left ear in both conditions. Except for electrode placement, identical stimulation protocols were used in both conditions. For both conditions, intensity was determined by the participants. After the electrode had been placed, stimulation began at 0 mA and gradually increased in increments of 0.1 mA until participants reported a moderate but tolerable level of discomfort, strong enough that they did not want to increase it, yet acceptable for 20 min of exposure. This self‐selected intensity was maintained throughout the session unless participants experienced excessive irritation or discomfort (*n* = 2 in each experiment). In such cases, the intensity was reduced to a comfortable level that was then maintained for the remainder of the session.

Transcutaneous VNS was delivered using a TENS ECO 2 (Schwa‐medico Inc.) stimulator with TENS OK electrode clamps. Stimulation was delivered to the left cymba concha, with the electrode positioned flush against the targeted area (tVNS condition) or clamped around the left earlobe (Control condition). Participants either received tVNS at a frequency of 25 Hz (beta frequency) with a pulse width of 200 μs (Experiment 1) or at a frequency of 55 Hz (gamma frequency) with a pulse width of 200 μs (Experiment 2). The specific beta and gamma frequencies were selected based on recent recording of OB responses to odor stimuli (Iravani et al. 2020, 2021; Nordén et al. 2024; Kay and Beshel 2010; Nordén et al. 2024; Yang et al. 2022).

### 1.3 Behavioral Measurements

#### 1.3.1 Olfactory Screening

Prior to initiating the experiment protocol, we ensured that all participants had a functional sense of smell using the Sniffin’ Sticks identification test (Burghart Messtechnik, Holm, Germany). The test consists of 16 felt‐tip pens, each containing a distinct odor that participants are tasked with identifying using four‐alternative forced‐choice cards. One point is awarded for each correct identification, yielding a total score between 0 and 16. We used an inclusion cut‐off of at least 9 correct identifications.

#### 1.3.2 Odor Discrimination

Odor discrimination was assessed using a modified version of the Sniffin’ Sticks discrimination set (Burghart Messtechnik, Holm, Germany). The standardized test consists of 16 triplets of felt‐tip pens containing distinct odors, two identical and one different. Participants are tasked with discriminating between the triplets and identifying the odd odor, and each correct response is scored as one point, yielding a total score between 0 and 16. In addition, to increase the test's variance and difficulty, we selected 8 of the target odors from the original 16 odor triplets that previous data has indicated as the most difficult to discriminate. These odors were then presented again in a modified triplet format where the original target odor was presented twice as a lure while the previously repeated lure odor was only presented once, thereby becoming the new target. This provided an extended discrimination task yielding a total score range of 0–24 with all odors validated for quality discrimination tasks (Kobal et al. 1996).

#### 1.3.3 Odor Intensity

Perceived odor intensity was assessed by presenting 10 mL each of 4 different odors in 240 mL amber glass bottles and asking participants to rate the perceived intensity using a labeled visual analog scale ranging from 0 (no sensation) to 100 (extremely intense). Odors were 1‐octen‐3‐ol (CAS 3391‐86‐4), geraniol (CAS 1066‐24‐1), guaiacol (CAS 90–05‐1), and 1‐butanol (CAS 71–36‐3). All odorants were diluted to 10% volume‐to‐volume in neat diethyl phthalate (CAS 84–66‐2).

#### 1.3.4 Visual Response Task

Because tVNS stimulation has been reported to enhance performance on attention and decision‐making tasks (Ridgewell et al. 2021), potentially confounding performance on the odor tasks, participants also performed a visual response task after the tVNS stimulation. In this task, the onset of each trial began with a visual presentation of a black fixation cross in the center of a white screen. At variable time intervals, a green circle appeared on the screen, and participants had to press a key as fast as they could upon detection of the circle. Faster responses (shorter response times) correspond to better performance. The task comprised 80 trials (5 of which were practice). To limit anticipatory responses or missed trials, all trials with a response time of less than 100 ms or longer than 1000 ms were deemed ineligible. Note that in Experiment 2, the result from one participant was excluded from all analyses of the visual response task due to less than 70% eligible responses, meaning that a total of 41 participants are included in the visual response analyses.

#### 1.3.5 Perceived Stimulation Intensity

To assess degree of discomfort, participants rated their average perceived intensity of stimulation per session, ranging from 0 (no sensation) to 100 (extremely painful) on a visual analog scale after the stimulation protocol.

### 1.4 Procedure

All participants underwent stimulation of both the cymba concha area (tVNS) and the earlobe (active control stimulation), separately in two sessions to limit potential carry‐over effects, resulting in a within‐subject design. The two sessions were held on different days, each lasting approximately 1.5 h, and separated by between 24 and 48 h. To minimize potential confounding effects on odor sensitivity, participants were instructed to abstain from smoking, eating, and drinking (except for water) for 1 h prior to the testing sessions. With the exception of stimulation type, all sessions were identical across experiments.

Each testing session began with assessment of baseline olfactory performance of odor discrimination and intensity ratings. Electrodes were then attached, stimulation intensity calibrated, and a 20‐min stimulation period was initiated. Throughout the stimulation period, participants watched the same video sequence from BBC nature show Planet Earth. The experimenter verbally assessed participants' comfort at two time points during the stimulation, once 5 min after onset and again at the halfway point. Following the stimulation period, participants completed the visual response task. After completion, participants' poststimulation olfactory performance was assessed using identical odor discrimination and intensity tasks as used at baseline. Lastly, participants reported the average ear stimulation intensity they perceived throughout the session.

### 1.5 Statistical Analysis

Effects of tVNS on olfactory functions were assessed using 2 × 2 repeated‐measures ANOVA (rm‐ANOVA) with the factors Treatment (tVNS vs. Control) and Time (prestimulation vs. poststimulation performance) for each measure separately. Effects of tVNS on the visual attention task were assessed using an rm‐ANOVA between the two Treatments. To assess potential differences between experiments, we performed an ANOVA with factors Treatment and Time as within‐subjects repeated‐measures and Frequency as between‐subjects measure. For all tests, a *p*‐value of 0.05 was used as the threshold for statistical significance. All analyses were performed using the JASP (version 0.95.4) statistical software. All data can be found on the Open Science Framework; link below under Data Sharing statement.

## 2 Results

### 2.1 Experiment 1—Beta Frequency Stimulation

#### 2.1.1 Odor Discrimination

We first assessed whether tVNS stimulation in beta frequency (25 Hz) modulated participants' odor discrimination performance using a 2 × 2 repeated‐measures ANOVA (rm‐ANOVA) with the factors Treatment (tVNS vs. Control) and Time (prestimulation vs. poststimulation performance). Twenty minutes of tVNS did not significantly alter discrimination performance, as indicated by the absence of a significant interaction between Treatment and Time, *F*(1,24) = 0.48, *p* = 0.49.

#### 2.1.2 Odor Intensity

**FIGURE 2:**
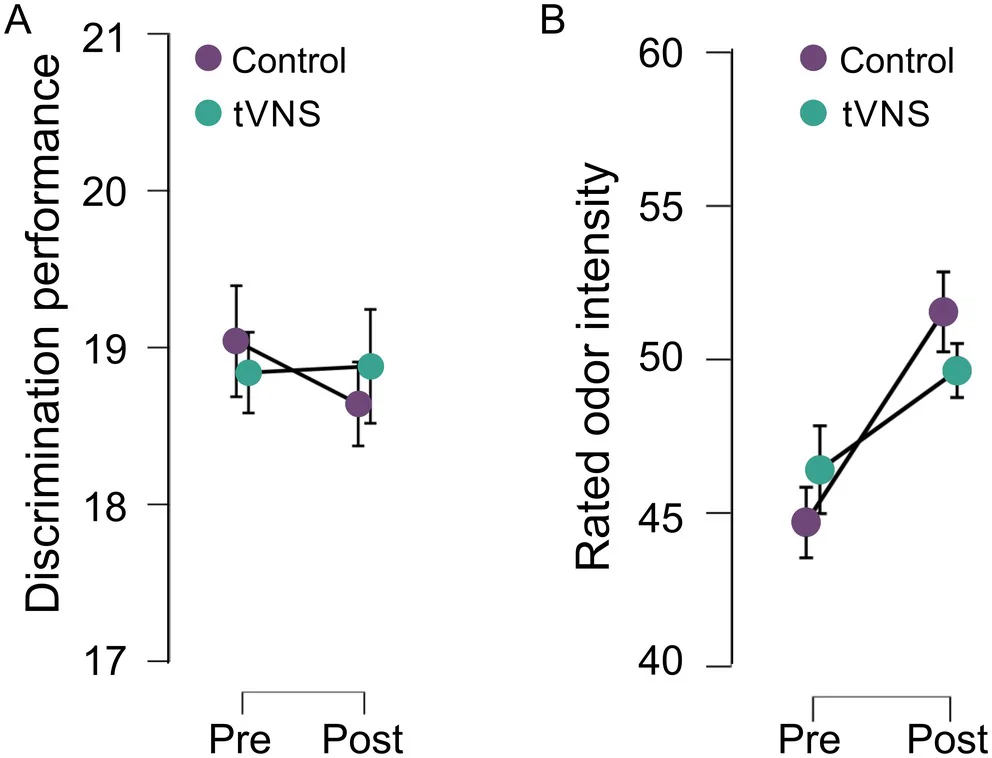
Results from Experiment 1 (beta frequency). (A) Mean discrimination performance. (B) Mean odor intensity rating. Error bars denote standard error of the mean.

We then examined whether odor intensity ratings were modulated by tVNS using the same rm‐ANOVA design. Similar to discrimination performance, there was no significant interaction between Treatment and Time for intensity ratings, *F*(1,24) = 2.67, *p* = 0.12 (Figure 2B). However, there was a significant effect of Treatment with intensity ratings increasing from pre‐ to poststimulation in both the tVNS, *t*(24) = 4.12, *p* < 0.0001, and the control condition, *t*(24) = 2.05, *p* < 0.026.

#### 2.1.3 Visual Attention

Finally, we determined whether tVNS modulated participants' performance on the visual attention task by examining differences between conditions using an ANOVA. There were no significant effects of Treatment on either reaction time, *F*(1,24) = 3.54, *p* = 0.07, or the number of correct trials, *F*(1,24) = 0.39, *p* = 0.54; see Supplementary Figure S1.

### 2.2 Experiment 2—Gamma Frequency Stimulation

#### 2.2.1 Odor Discrimination

**FIGURE 3:**
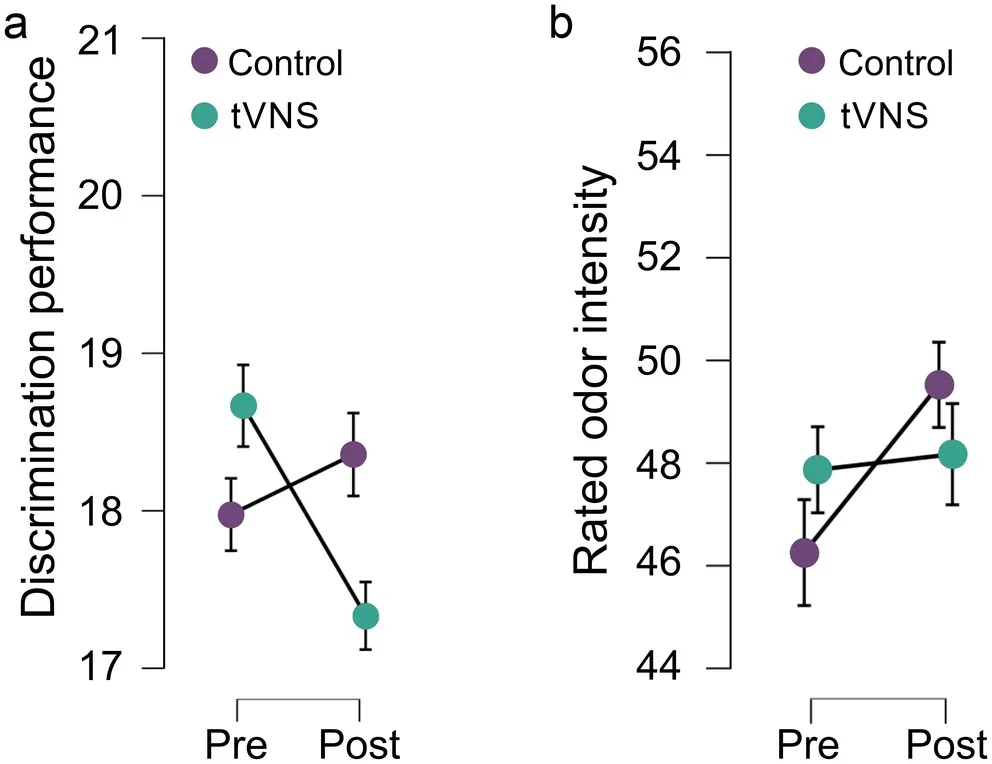
Results from Experiment 2 (gamma frequency). (a) Mean discrimination performance. (b) Mean odor intensity ratings. Error bars denote standard error of the mean.

We assessed the effect of gamma frequency tVNS (55 Hz) on odor discrimination using the same procedure as in Experiment 1 and found a significant interaction effect between Treatment and Time, *F*(1,41) = 15.89, *p* < 0.001 (Figure 3a).

#### 2.2.2 Odor Intensity

We then examined potential tVNS effects on odor intensity ratings. There was no significant interaction between Treatment and Time, *F*(1,41) = 2.66, *p* = 0.11 (Figure 3b).

#### 2.2.3 Visual Attention

Finally, we assessed whether tVNS in the gamma band modulated participants' performance on the attention task. As for Experiment 1, we determined potential differences on attention‐related parameters between Treatment conditions with an ANOVA. There was no significant difference between conditions for either reaction time, *F*(1,40) = 1.92, *p* = 0.17, or number of correct trials, *F*(1,40) = 0.53, *p* = 0.47; see Figure S2.

#### 2.2.4 Perceived Stimulation Intensity

Given that participants were able to set their stimulus intensity, we finally examined whether either the selected current (mA) or the self‐reported treatment intensity during the tVNS session was correlated with the effect of treatment (post–prestimulation). There was no significant correlation between odor discrimination effect and selected current, *r*(41) = 0.003, *p* = 0.96, nor between odor discrimination effect and perceived stimulation intensity, *r*(41) = −0.056, *p* = 0.73. Similarly, no significant correlation was observed between odor intensity ratings and selected current, *r*(41) = 0.14, *p* = 0.39, nor odor intensity ratings and perceived stimulation intensity, *r*(41) = 0.20, *p* = 0.21. However, as expected, there was a significant positive relationship between selected current and perceived treatment intensity, *r*(41) = 0.50, *p* < 0.001.

### 2.3 Comparison Between Experiment 1 and 2

Finally, we assessed potential differences in tVNS effects on discrimination performance and rated perceived odor intensity differences between the two experiments. There was a significant three‐way interaction for discrimination performance, *F*(1,65) = 8.46, *p* < 0.005. However, there was no significant three‐way interaction for perceived odor intensity, *F*(1,65) = 0.05, *p* < 0.82.

There is a difference in sample size between the two experiments, thereby raising the question of whether the lack of effects in Experiment 1 is due to insufficient statistical power. To assess this, we inflated the sample by bootstrapping the repeated measures ANOVAs in Experiment 1 (1000 resamples) which confirmed that the Treatment × Time interaction effect sizes were nonsignificant for both odor discrimination, *p* > 0.49, *η* ^*2*^ = 0.02, and odor intensity, *p* > 0.11, *η* ^*2*^ = 0.10.

## 3 Discussion

We investigated whether tVNS modulates human olfactory bulb function, assessed through changes in odor discrimination and odor intensity perception. Beta‐frequency tVNS (25 Hz), a frequency associated with top‐down feedback processing in the human OB (Kay and Beshel 2010; Nordén et al. 2024; Yang et al. 2022), did not produce measurable effects on odor discrimination. However, gamma‐frequency tVNS (55 Hz), a frequency corresponding to the endogenous oscillatory rhythm of the OB during active odor processing (Iravani et al. 2020), significantly reduced discrimination performance. This dissociation, as demonstrated by a significant difference between experiments, is consistent with the hypothesis that the effects of tVNS on the OB are frequency‐specific and dependent on the intrinsic oscillatory dynamics of the targeted neural circuits. Odor intensity ratings increased modestly in both experiments, with a somewhat stronger effect following gamma‐frequency stimulation, though the Treatment × Time interaction did not reach significance in either case.

The reduction in odor discrimination performance following gamma‐frequency tVNS aligns with the mechanistic prediction derived from animal electrophysiology. In the foundational rodent work by García‐Díaz et al. (1984), VNS excited inhibitory interneurons in the periglomerular layer of the OB, producing decreased discharge rates in approximately half of recorded neurons. This increased periglomerular inhibition would be expected to reduce the contrast between competing odor representations at the level of mitral and tufted cell output, thereby impairing the ability to distinguish among similar odorants. Our data are consistent with this account: acute tVNS at a frequency that engages the OB's endogenous processing rhythm produced a measurable decrease in discrimination performance, while the control condition did not. Our data cannot, however, conclusively determine whether the observed behavioral change reflects modulation specifically within the periglomerular layer, as opposed to broader changes in excitation‐inhibition balance across the OB circuitry or in downstream olfactory cortical areas. Nonetheless, several mechanisms remain compatible with the present data, including altered excitation–inhibition balance within OB circuitry, changes in gain modulation of mitral and tufted cell output, and broader neuromodulatory state changes; modulation of inhibitory interneuron activity within the OB is, in our view, the candidate most directly supported by the animal literature, but the present data cannot differentiate between these accounts. We note that the relationship between OB inhibition and discrimination behavior is not monotonic: both increasing and decreasing granule cell inhibition alter discrimination performance in rodents (Gschwend et al. 2015; Nunes and Kuner 2015), and whereas learning‐related, stimulus‐locked recruitment of inhibitory circuits sharpens odor representations (Abraham et al. 2010), exogenously driven inhibition that is not temporally coupled to sensory input may instead degrade the contrast between competing representations.

It is worth noting that the direction of our behavioral effect, a decrease in discrimination, appears to diverge from some prior reports suggesting that VNS improves sensory discrimination. In the rodent literature, increased inhibitory interneuron activity in the OB has typically been interpreted as enhancing signal‐to‐noise ratios by suppressing background activity, thereby sharpening odor representations and improving discrimination (Abraham et al. 2010; Yokoi et al. 1995). However, this interpretation rests primarily on chronic or repeated stimulation paradigms in which activity‐dependent plasticity may refine circuit function over time. In contrast, our paradigm assessed performance immediately following a single 20‐min stimulation session, a context in which the acute increase in inhibition may overwhelm the system rather than optimize it. Biological systems often exhibit a transient decline in function before adaptive strengthening emerges over time. For example, intense training of a muscle group weakens performance when assessed immediately after exercise, but over the long term produces increased strength through compensatory activity‐dependent plasticity, so‐called supercompensation. This distinction between acute disruption and longer‐term enhancement is well established in other domains of neural plasticity; for example, repetitive transcranial magnetic stimulation of motor cortex transiently disrupts performance when assessed immediately after stimulation but can enhance function with repeated application over days or weeks (Ridding and Rothwell 2007). Whether a similar trajectory applies to tVNS‐induced modulation of OB circuits, such that repeated 55 Hz frequency stimulation could ultimately improve discrimination after a rest period, remains an open question that warrants investigation with multi‐session protocols.

Our findings can be situated within the small but growing body of human studies examining VNS effects on olfactory function. Maharjan, Wang, et al. (2018) reported that high‐frequency (80 Hz) noninvasive auricular VNS improved supra‐threshold olfactory performance in healthy adults, while low‐frequency (10 Hz) stimulation had no effect. While both that study and ours converge on the conclusion that stimulation frequency is a critical parameter, the direction of the behavioral effects differs. Maharjan and colleagues observed improvement, whereas we observed impairment. Several factors may account for this discrepancy. First, the olfactory measures differed; Maharjan and colleagues used a supra‐threshold intensity discrimination test whereas we assessed odor quality discrimination using a triplet forced‐choice paradigm. These tasks likely engage partially distinct neural substrates, with intensity discrimination relying more on peripheral gain mechanisms and quality discrimination depending on OB lateral inhibitory circuits. Second, the stimulation frequencies differed (80 Hz vs. 55 Hz), and it remains possible that these engage partially different neuromodulatory pathways or different populations of OB interneurons. Third, Maharjan et al. assessed performance during stimulation, whereas we assessed performance after stimulation had ceased, introducing differences in the temporal relationship between neural modulation and behavioral measurement. Weise et al. (2025) applied high frequency tVNS (80 Hz) stimulation of the vagus nerve in patients with post‐COVID‐19 olfactory dysfunction and demonstrated improved odor discrimination in the patient group, but not in normosmic controls where a ceiling effect may have obscured potential changes. This pattern suggests that the baseline state of OB circuits, whether intact or compromised, may critically determine the behavioral direction of VNS effects, a possibility consistent with the broader neuromodulation literature in which stimulation effects are often state‐dependent (Silvanto et al. 2008).

By contrast, studies using lower‐frequency or invasive VNS at standard clinical settings (20–30 Hz) have generally failed to show behavioral improvements in olfactory function. Kirchner et al. (2004) found no changes in psychophysical olfactory measures in epilepsy patients receiving implanted VNS, despite observing altered olfactory event‐related potentials. Similarly, Sperling et al. (2011) reported no changes in olfactory thresholds or discrimination in depression patients on chronic VNS therapy, even as gustatory perception was significantly altered. These null behavioral findings at low stimulation frequencies are consistent with our Experiment 1 result, where 25 Hz tVNS produced no measurable change in discrimination, and reinforce the conclusion that stimulation frequency is a critical determinant of whether vagus input reaches or modulates OB circuits.

The modest increase in odor intensity ratings observed in both experiments, though not reaching significance as a Treatment × Time interaction, deserves comment. Perceived odor intensity increased from pre‐ to poststimulation in both the tVNS and control conditions, suggesting a general sensitization or practice effect, though the numerical increase was somewhat larger following tVNS. This pattern is broadly consistent with past findings that high‐frequency median nerve stimulation suppressed odor intensity ratings (Maharjan, Peng, and Cakmak 2018), supporting the general principle that peripheral nerve stimulation can modulate perceived sensory intensity through engagement of central gain‐control mechanisms. However, the absence of a significant interaction in our data precludes strong conclusions about whether tVNS specifically modulates intensity perception beyond any nonspecific effects of the experimental procedure.

Several methodological limitations warrant consideration. Direct comparisons between the two experiments demonstrated a significant difference. However, the two stimulation frequencies were tested in separate participant groups with a difference in sample size. This raises the question of whether the null result at 25 Hz reflects a true absence of effect or insufficient statistical power. While we cannot definitively rule out a power limitation, additional bootstrap analyses suggested that even with a substantially larger sample, the 25 Hz condition would be unlikely to yield a significant Treatment × Time interaction. A within‐subjects design crossing both frequencies would provide a stronger test of the frequency‐specificity claim but was not deemed feasible given the potential risk of carryover effects from repeated stimulation sessions. In addition, without direct measures of OB activity, broader neuromodulatory influences cannot be fully ruled out. However, global shifts in core cognitive processes are unlikely to account for these highly specific odor discrimination effects.

Further, our data do not demonstrate oscillatory entrainment of OB circuits, and the degree to which the temporal structure of tVNS pulse trains survives the polysynaptic route from auricular afferents to the OB is unknown; the match between stimulation frequency and endogenous OB rhythms motivated our frequency selection but remains hypothetical as a mechanism. An alternative locus for the frequency specificity is the brainstem itself, including the NTS–locus coeruleus pathway. We consider a simple differential‐recruitment account unlikely, both because rodent recordings show that VNS frequency alters the timing rather than the amount of driven locus coeruleus activity (Hulsey et al. 2017), and because the behavioral effect was confined to odor discrimination while visual attention and odor intensity were unaffected. However, frequency‐dependent temporal patterning of neuromodulatory input to olfactory structures cannot be excluded as a contributing mechanism.

Moreover, our active control condition, earlobe stimulation, warrants scrutiny. The earlobe is conventionally used as a sham site in tVNS research because it is considered to lack vagus innervation (Peuker and Filler 2002), and functional neuroimaging studies have confirmed that earlobe stimulation does not activate the NTS or locus coeruleus (Frangos et al. 2015; Yakunina et al. 2017). However, the earlobe is innervated by branches of the greater auricular nerve and possibly the auriculotemporal nerve, and there is a debate of its suitability as a control area (Keute et al. 2018; Rangon 2018). If earlobe stimulation produced any partial activation of vagus or trigeminal pathways in our paradigm, this would bias our results toward the null by reducing the contrast between conditions, meaning that the true effect of tVNS on discrimination could be larger than what we observed. With that said, the significant Treatment × Time interaction in Experiment 2 demonstrates that the two stimulation sites produced measurably different behavioral outcomes, supporting the validity of the earlobe as a control in this context. Furthermore, although the placement of the electrode in the tVNS condition is intended to target the auricular branch of the vagus nerve in the cymba concha, the precise area of stimulation cannot be fully controlled. Thus, while the stimulation is intended to primarily target the auricular branch of the vagus nerve, some activation of nearby nerve fibers cannot be entirely excluded. In addition, while the lack of a tVNS effect on the visual attention task suggests that the impact of tVNS on odor discrimination is not due to a general enhancement of performance, it remains possible that the mechanism leading to altered odor discrimination also affects performance for other attention tasks (Ridgewell et al. 2021).

Our findings demonstrate that noninvasive tVNS can modulate human olfactory processing at a stimulation frequency matched to the endogenous gamma rhythm of the olfactory bulb, but not at a frequency matched to its beta rhythm. This extends prior demonstrations of frequency‐dependent VNS effects on olfaction (Maharjan, Wang, et al. 2018; Weise et al. 2025) by grounding frequency selection in the measured oscillatory dynamics of the human OB and provides behavioral evidence for a putative vagus‐OB pathway in humans.

The selective effect of gamma‐frequency stimulation, combined with the null result at beta frequency, supports the principle that neuromodulatory interventions targeting the OB must be tuned to the intrinsic oscillatory dynamics of the target circuits.

Whether the acute disruption of discrimination observed here can be harnessed therapeutically through repeated stimulation protocols that promote long‐term plasticity remains a critical question for future work. Multi‐session studies examining the time course of tVNS effects, ideally combining behavioral measures with neuroimaging of OB activity, will be essential to determine whether tVNS can serve as a viable tool for olfactory rehabilitation in clinical populations with compromised OB function.

## Author Contributions

**Anja L. Winter:** writing – original draft, visualization, writing – review and editing, formal analysis. **Andrea Aejmelaeus‐Lindström:** writing – review and editing, visualization, resources. **Kristen Gregory:** methodology, data curation, project administration. **Gregory Francis:** conceptualization, writing – review and editing, methodology, resources. **Cristina Jaen:** formal analysis, data curation, writing – review and editing. **Pamela Dalton:** writing – review and editing, supervision, methodology. **Evelina Thunell:** methodology, writing – review and editing. **Artin Arshamian:** investigation, writing – review and editing, methodology, project administration. **Johan N. Lundström:** conceptualization, funding acquisition, writing – review and editing, methodology, supervision, formal analysis, project administration, resources.

## Funding

Funding was provided by a donation from Stiftelsen Bygg‐Göta för Vetenskaplig forskning and a grant from the Swedish Research Council (VR 2021‐02700), both awarded to J.N.L.

## Conflicts of Interest

The authors declare no conflicts of interest.

## Supporting information


**Figure S1:** Experiment 1—25 Hz. (A) Mean response times for each individual, seperated by condition. Line between circles in left panel indicate same participant. Middle box‐plot show group mean as solid horizontal line, box as lower and upper quartile values, and whiskers range of 95% of values. Right panel show distribution for each condition with overlap marked with brown color. (B) Number of correct trials for each individual, seperated by condition. Line between circles in left panel indicate same participant. Middle box‐plot show mean as solid horizontal line, box as lower and upper quartile values, and whiskers range of 95% of values. Right panel show distribution for each condition with overlap marked with brown color. In all panels, green color indicate vagus nerve stimulation (VNS) condition and orange color indicate ear lobe (Control) stimulation condition.
**Figure S2:** Experiment 2—55 Hz. (A) Mean response times for each individual, seperated by condition. Line between circles in left panel indicate same participant. Middle box‐plot show group mean as solid horizontal line, box as lower and upper quartile values, and whiskers range of 95% of values. Right panel show distribution for each condition with overlap marked with brown color. (B) Number of correct trials for each individual, seperated by condition. Line between circles in left panel indicate same participant. Middle box‐plot show mean as solid horizontal line, box as lower and upper quartile values, and whiskers range of 95% of values. Right panel show distribution for each condition with overlap marked with brown color. In all panels, green color indicate vagus nerve stimulation (VNS) condition and orange color indicate ear lobe (Control) stimulation condition.

## Data Availability

All data can be found on the Open Science Framework (OSF) at https://osf.io/aurd8/overview?view_only=0b61b28f5f7a42ada3b6ce50c779a22c.
