# Supplementary material for: Transcutaneous Vagus Nerve Stimulation at Olfactory Bulb‐Relevant Frequencies Modulates Human Olfactory Function
Source: Psychophysiology. 2026 Sep 22;63(9):e70408. doi: 10.1111/psyp.70408 (PMC13598272; doi:10.1111/psyp.70408)
Supplement: Supplementary file 1 [file PSYP-63-e70408-s001.docx]

# Supplementary figures

***Supplementary Figure 1. Experiment 1 - 25Hz. A.*** ***Mean response times*** *for each individual, seperated by condition. Line between circles in left panel indicate same participant. Middle box-plot show group mean as solid horizontal line, box as lower and upper quartile values, and whiskers range of 95% of values. Right panel show distribution for each condition with overlap marked with brown color.* ***B.*** ***Number of correct trials*** *for each individual, seperated by condition. Line between circles in left panel indicate same participant. Middle box-plot show mean as solid horizontal line, box as lower and upper quartile values, and whiskers range of 95% of values. Right panel show distribution for each condition with overlap marked with brown color. In all panels, green color indicate vagus nerve stimulation (VNS) condition and orange color indicate ear lobe (Control) stimulation condition.*

***Supplementary Figure 2. Experiment 2 - 55Hz. A.*** ***Mean response times*** *for each individual, seperated by condition. Line between circles in left panel indicate same participant. Middle box-plot show group mean as solid horizontal line, box as lower and upper quartile values, and whiskers range of 95% of values. Right panel show distribution for each condition with overlap marked with brown color.* ***B.*** ***Number of correct trials*** *for each individual, seperated by condition. Line between circles in left panel indicate same participant. Middle box-plot show mean as solid horizontal line, box as lower and upper quartile values, and whiskers range of 95% of values. Right panel show distribution for each condition with overlap marked with brown color. In all panels, green color indicate vagus nerve stimulation (VNS) condition and orange color indicate ear lobe (Control) stimulation condition.*
